# Noninvasive Techniques for Blood Pressure Measurement Are Not a Reliable Alternative to Direct Measurement: A Randomized Crossover Trial in ICU

**DOI:** 10.1155/2014/353628

**Published:** 2014-01-30

**Authors:** Sara Ribezzo, Eleonora Spina, Stefano Di Bartolomeo, Gianfranco Sanson

**Affiliations:** ^1^School of Nursing, University of Trieste, 34100 Trieste, Italy; ^2^Intensive Care Unit, University Hospital of Trieste, 34100 Trieste, Italy; ^3^Department of Anesthesia 1, University Hospital of Udine, 33100 Udine, Italy; ^4^Emilia-Romagna Regional Agency for Health and Social Care, 40100 Bologna, Italy

## Abstract

*Introduction.* Noninvasive blood pressure (NIBP) monitoring methods are widely used in critically ill patients despite poor evidence of their accuracy. The erroneous interpretations of blood pressure (BP) may lead to clinical errors. *Objectives.* To test the accuracy and reliability of aneroid (ABP) and oscillometric (OBP) devices compared to the invasive BP (IBP) monitoring in an ICU population. *Materials and Methods.* Fifty adult patients (200 comparisons) were included in a randomized crossover trial. BP was recorded simultaneously by IBP and either by ABP or by OBP, taking IBP as gold standard. *Results.* Compared with ABP, IBP systolic values were significantly higher (mean difference ± standard deviation 9.74 ± 13.8; *P* < 0.0001). Both diastolic (−5.13 ± 7.1; *P* < 0.0001) and mean (−2.14 ± 7.1; *P*=0.0033) IBP were instead lower. Compared with OBP, systolic (10.80 ± 14.9; *P* < 0.0001) and mean (5.36 ± 7.1; *P* < 0.0001) IBP were higher, while diastolic IBP (−3.62 ± 6.0; *P* < 0.0001) was lower. Bland-Altman plots showed wide limits of agreement in both NIBP-IBP comparisons. *Conclusions.* BP measurements with different devices produced significantly different results. Since in critically ill patients the importance of BP readings is often crucial, noninvasive techniques cannot be regarded as reliable alternatives to direct measurements.

## 1. Introduction

Arterial blood pressure (BP) is one of the most frequently measured parameters in clinical practice, as many diagnostic and therapeutic decisions are based on this measure. The reliability of BP measurements is particularly important in critically ill patients, who often need frequent or continuous BP monitoring to establish or reassess their treatments. Different systems derive systolic (SAP), diastolic (DAP), and mean (MAP) arterial pressure parameters based on different physical events. These events are the Korotkoff tones for aneroid manometers (ABP), the maximal oscillations of a cuff pressure curve for oscillometric devices (OBP), and direct electronic measurement for invasive arterial blood pressure (IBP).

In some clinical settings the ABP technique with manual aneroid manometers (mercury manometers having been banned a few years ago) remains the method of choice for BP measurement [1], despite its inaccuracy in the absence of frequent recalibration [2]. IBP monitoring (arterial cannulation with continuous pressure transduction and waveform display) is instead the reference standard for BP monitoring in intensive care unit (ICU) patients. However, it is expensive, carries an increased risk of complications, and requires more clinical expertise than noninvasive monitoring [3]. Consequently, noninvasive BP (NIBP) monitoring systems, comprising ABP and OBP, are often preferred in the ICU. The auscultatory technique is seldom used in critically ill patients, except in some situations like emergencies or transports where it may be the only available method. OBP monitoring devices are also mainly used for out-of-office BP measurements, but they offer several advantages compared to aneroid instruments, such as the possibility of automatic measurements or the direct measurement of MAP.

Unfortunately, NIBP monitoring is influenced by factors related to the procedure, to the instruments themselves, and to interobserver variability [4]. Because noninvasive methods may not be sufficiently accurate in critically ill patients, leading to erroneous interpretations of BP and possible errors in clinical decisions [5], there is a need for validation studies comparing the accuracy and precision of NIBP and IBP monitoring [6].

The objective of this study is therefore to compare invasive arterial blood pressure with noninvasive blood pressure measurements, considering that the measurements with invasive method reliably reflect the actual value of BP.

## 2. Materials and Methods 

### 2.1. Study Design and Patient Selection

This is a randomized crossover clinical trial performed to test the accuracy and reliability of ABP and OBP compared with invasive arterial blood pressure (IBP) monitoring. The study was carried out from July to December 2012 in the General ICU of the “Azienda Ospedaliero-Universitaria” Hospital of Trieste, Italy. The ICU has 13 beds and admits about 800 patients a year. During the study period, 50 patients aged between 18 and 92 years with a radial artery catheter were included in the study. The caregivers took the decision to place an intra-arterial catheter without any influence from the researchers. Because the other trial interventions were noninvasive and did not interfere with usual patients care, no informed consent was required according to the hospital authorities.

Exclusion criteria were the presence of a different arterial line (e.g., femoral or brachial), any contraindication to cuff application/inflation (e.g., arm injuries or wounds), Glasgow Coma Scale ≥12, and the presence of arrhythmias. For every patient, main diagnosis and ongoing infusion of vasoactive drugs were also recorded.

### 2.2. BP Measurements

BP measurements were performed by 3 critical care nurses. In order to obtain accurate and consistent readings, each nurse had been trained to follow a standardized BP recording method [1] before starting the trial. As part of the training, the nurses measured the blood pressure by the auscultatory method on 10 healthy volunteers to verify their interobserver consistency (±5 mmHg).

During the study, the BP was measured 4 consecutive times per patient on the same arm [7], twice by sphygmomanometer and twice by oscillometric device, in both cases simultaneously with IBP monitoring. A 5-minute interval separated one assessment from the other to avoid the compression applied to the arm possibly affecting the following measurements. The type and sequence of BP measurements (IBP, ABP, and OBP) were assigned randomly. Four data collection forms for each patient were prepared in different sealed envelopes with all possible combinations of both the BP device sequence (OBP-ABP-OBP-ABP or ABP-OBP-ABP-OBP) and the name of the nurse in charge of the IBP, OBP, or ABP measurement. Before starting each patient's BP measurement procedure, one of the nurses blindly chose one of the four envelopes containing the forms. The nurses that measured BP either by aneroid sphygmomanometers or oscillometric devices were blinded to the values measured by the invasive technique, as they were recorded one second before the nurse started the inflation of the cuff. The monitor used for radial IBP and brachial OBP measurement was an IntelliVue MP70 (Royal Philips Electronics, the Netherlands). ABP was measured with a well-calibrated DuraShock integrated aneroid sphygmomanometer (Welch Allyn, Inc., USA), DS44-10 (small adult cuff size), DS44-11 (adult cuff size), and DS44-12 (large adult cuff size).

A strict study protocol prescribed that the IBP-designated nurse calibrated the arterial line by (1) positioning the transducer at the level of the patient's 4th intercostal space at the midaxillary line, (2) regulating the pressure reading to zero, (3) inspecting the tubing and transducer to ensure absence of kinking or air bubbles, and (4) flushing the tube and performing a “fast flush” test to verify the presence of a normal arterial waveform, a natural frequency between 16 and 25 Hz, and a damping coefficient between 0.5 and 0.9*ζ*.

Given that the current literature reports risks of inaccuracy of auscultatory BP measurement related to both observer and methodological errors, the following precautions were taken. We avoided digit preferences, and SAP and DAP values were rounded to the closest 2 mmHg. Cuff deflation was standardized at 2 mmHg per second. An appropriately sized cuff was chosen following a measurement of arm circumference (at the midpoint between the acromion and the humeral epicondyle). The nurse was positioned so as to be able to see the dial of the manometer perpendicularly and at eye level. In case of persistence of the fifth Korotkoff tone, attenuation of the fourth tone was acquired as a measure of DAP. To prevent possible bias resulting from “white coat hypertension” effect, we included only patients sedated or with reduced level of consciousness (Glasgow Coma Scale <12). To calculate the MAP from the values obtained by the sphygmomanometer we used the formula (SAP + 2DAP)/3.

### 2.3. Statistical Analysis

Statistical analysis was performed using the statistical software Stata SE version 10. Continuous variables (age, BP measures) are displayed as mean ± standard deviation (SD) and median. Nominal variables (gender, arrhythmias, and use of drugs) are displayed as number and percentage and analysed using 2 × 2 contingency tables and Fisher's exact test. Paired *t*-test was used for comparisons between means. The correlation between the SAP, DAP, and MAP values of the ABP/IBP and OBP/IBP comparisons was investigated with linear regression, Pearson's correlation coefficient, and a Bland-Altman chart [8]. The accuracy of BP measurement comparisons was estimated according to the British Hypertension Society (i.e., a minimum percentage of readings must be within 5, 10, and 15 mmHg [9] and the Association for the Advancement of Medical Instrumentation (i.e., average differences no greater than ±5 mmHg) and SD no greater than 8 mmHg) [10]. The criterion for statistical significance was *P* < 0.05.

The sample size was determined to detect, with a probability of a type I error of 0.05 and a type II error of 0.2 (paired *t*-test), a between-technique minimum difference in mean SAP of approximately 6 mmHg, in case of an SD of ±15 mmHg, and a 4 mmHg difference in case of an SD of ±10 mmHg (range of SD plausible values taken from previous literature).

## 3. **Results**

### 3.1. General Characteristics of the Studied Patients

There were 32 females (64%) and 18 males (36%). Their mean age was 65.3 ± 16 years (median 73). The causes of admission to the ICU were acute ischemic or haemorrhagic stroke (21; 42%), severe sepsis or septic shock (13; 26%), major polytrauma (6; 12%), respiratory failure (4; 8%), postoperative complications (3; 6%), and postcardiac arrest syndrome (3; 6%). Twenty-six measurements for each comparison were collected while vasoactive drugs (norepinephrine, 0.3–2.9 mg/h) were administered.

### 3.2. Blood Pressure Measurements

**Figure 1:**
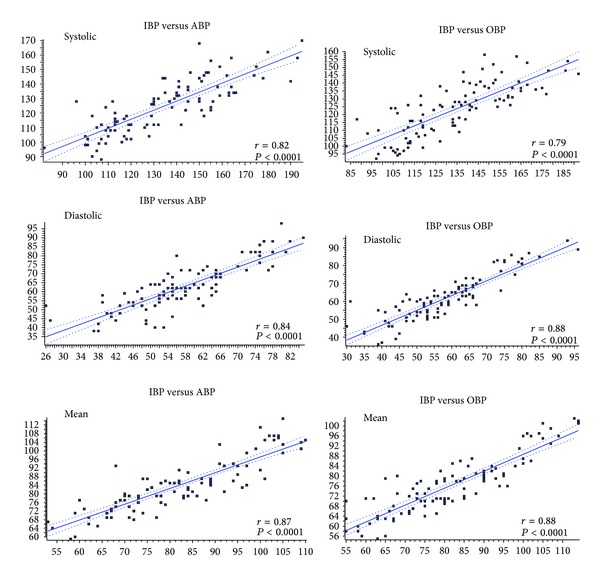
Scatterplot and correlation between systolic, diastolic, and mean blood pressure in comparisons between direct-invasive (IBP) and, respectively, auscultatory-aneroid (ABP) and oscillometric automated (OBP) methods.

Overall 50 cycles of 4 BP measurements were performed and 200 comparisons collected (100 comparisons between ABP and IBP and 100 between OBP and IBP). Based on all measurements the correlations between ABP and OBP devices and IBP measurements were highly significant (all *P* < 0.001) (Figure 1).

**Figure 2:**
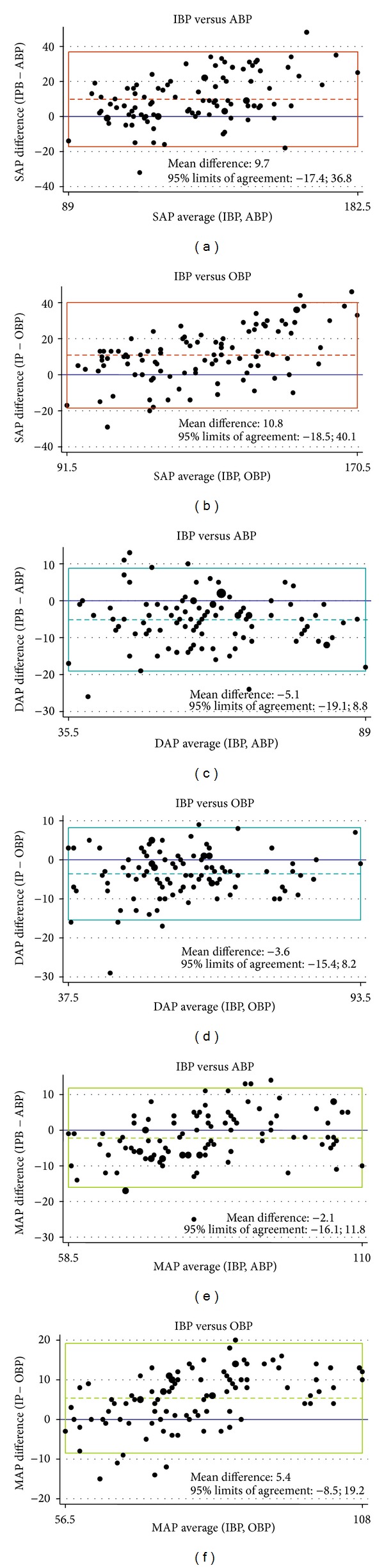
Bland-Altman analysis of the agreement between systolic (SAP), diastolic (DAP), and mean (MAP) arterial pressure in comparisons between direct-invasive (IBP) and, respectively, auscultatory-aneroid (ABP) and oscillometric automated (OBP) methods. The dashed line represents the mean bias; the upper and lower limits of the box represent the 1.96 ± SD limits of agreement.

Accuracy was defined as the agreement between the concomitant IBP and ABP/OBP measurements using the method of Bland-Altman (Figure 2). The Bland-Altman analysis indicated that the mean differences between NIBP and IBP were, respectively, 9.74 and 10.8 (systolic pressure), −5.1 and −3.6 (diastolic pressures), and −2.1 and 5.4 (mean arterial pressure), when evaluated by ABP and OBP, respectively. The Bland-Altman plots showed also that both ABP and OBP tended to underestimate SAP when it was in the higher range and to overestimate it if it was in the lower range. NIBP readings seemed to overestimate MAP for low BP values, whereas the oscillometric method underestimated high MAP values.

The averages of all systolic, diastolic, and mean blood pressure measurements obtained in the study population by the noninvasive devices and by IBP monitoring are reported in Table 1. SAP values assessed by NIBP devices were significantly lower compared with the values from IBP, and DAP values were significantly higher when measured by NIBP rather than by the IBP technique. Compared to IBP monitoring, MAP obtained with ABP was higher, while it was lower when measured with OBP. Applying the AAMI criteria, the agreement between the two methods (ABP/IBP and OBP/IBP) was confirmed for both diastolic and mean but not for systolic readings.

We also compared the two methods in terms of percentages of readings that varied by ≤20, ≤15, ≤10, and ≤5 mmHg (Table 2). According to the BHS protocol, very good agreement was achieved only by DAP in the IBP/OBP comparison. SAP showed the worst agreement for both comparisons, with almost a quarter of measurements exceeding 20 mmHg of disagreement.

## 4. Discussion

To date, there are different methods available to measure arterial systolic, diastolic, and mean BP, which rely on the detection of different physical events. IBP monitoring is commonly used in the ICU and normally consists of a column of fluid directly connecting an arterial catheter to a pressure transducer, which converts the pressure waveform into an electrical signal. This signal is processed, amplified, converted, and displayed as BP value and graphic waveform. In the absence of technical errors (e.g., kinking, bubbles or clots in the cannula/tubing, and wrong positioning of the transducer) IBP is considered the golden standard for BP measurement in the ICU. IBP provides several advantages over less invasive methods: it allows quick and easy blood sampling, it ensures close monitoring through continuous beat-to-beat BP measurement, its readings remain reliable in obese, neonate, burned, haemodynamically unstable, or arrhythmic patients, and it generates waveforms that allow pulse contour analysis.

The auscultatory method prescribes instead that a cuff be placed around the upper arm, inflated above systolic pressure to occlude the brachial artery, and subsequently slowly deflated. The restoration of blood flow is associated with the detection of Korotkoff sounds by a stethoscope over the artery. The first clearly audible sound corresponds to SAP (Korotkoff's phase 1) and the last audible sound (phase 5) to DAP. In situations in which the fifth sounds are audible even after complete deflation of the cuff, the fourth sound can be used as DAP. Previous research has found that this method tends to give lower values of SAP and higher of DAP when compared with the true intra-arterial pressure [1]; our results confirm these findings.

Oscillometric devices record the oscillations of pressure in a sphygmomanometer cuff during its progressive deflation; the maximal detected oscillation corresponds to MAP, while SAP and DAP are estimated according to various empirical algorithms usually not disclosed by manufacturers that may result in dramatically different accuracy levels [11]. Moreover, the amplitude of the oscillations may depend on factors other than BP, that is, the stiffness of the arteries and the site of measurement, because in more distal arteries SAP tends to increase and DAP to decrease [12]. Additionally, since the cuffs deflate at a manufacturer-specific speed that assumes a regular pulse, OBP is unreliable in arrhythmic patients. A large number of studies have demonstrated that OBP measurements obtained by wrist, finger, or brachial oscillometric devices do not achieve adequate accuracy in either adult or paediatric critically ill patients [13–21]. Only in a few studies from paediatric populations were the BP measurements obtained by wrist devices consistent with those recorded by IBP [22, 23].

However, noninvasive methods are still widely used in the ICU [6]. Our study shows, in accordance with previous investigations [24–26], that noninvasive methods may be inaccurate among critically ill patients and lead to erroneous interpretations of BP. In particular, our data show that noninvasive methods are extremely imprecise in measuring SAP. This may have negative consequences for clinical decisions and on the calculation of various scores based on it. We found a better accuracy for MAP measurement by the auscultatory technique. The accuracy was instead lower for MAP measured by the oscillometric method. This finding was unexpected because MAP is automatically calculated by the oscillometric device independently of SAP and DAP, whereas the MAP measured by sphygmomanometer is worked out by the operator basing on SAP and DAP values according to a formula and therefore may be more influenced by the inaccuracy of the latter values [12, 27].

Overall, according to the BHS criteria, we found a large percentage of readings outside the range of acceptable agreement. This may result in greater risks of erroneous clinical decisions—for example, unnecessary use of inotropic support and blood transfusions in hypotensive patients or, conversely, delayed antihypertensive treatment in hypertensive patients [13, 19, 28]. Unfortunately, the literature is not consistent about the range of accuracy that can be considered acceptable in critically ill patients. In anesthetized patients, Gibbs et al. [29] suggest that differences greater than 10 mmHg should be regarded as clinically relevant and that they become clinically unacceptable in excess of 20 mmHg. If we accept this definition, only a very few measurements of NIBP did not show clinically relevant differences from the gold standard. However, it has been pointed out that the clinical relevance of BP discrepancies should be gauged based on the overall haemodynamic situation of the ICU patient. A difference of more than 10 mmHg in a patient with a MAP <60 mmHg is clinically more relevant than the same difference in a patient with a MAP of 100 mmHg [13].

In accordance with previous studies [18, 20, 28], our data also showed that the average difference detected when comparing noninvasive to direct methods was not constant and that the discrepancies varied across the methods and the types of BP (i.e., SAP, MAP, and DAP).

In a study from Takci et al. [20] where IBP monitoring was compared to OBP in 27 critically ill preterm infants, oscillometric MAP was found to be significantly higher in the presence of hypotension (*P* < 0.05), while no statistically significant difference was shown for normal or high pressure values. Holt et al. [18] compared IBP with OBP and sphygm/Doppler ultrasound BP measurements in 40 paediatric ICU patients and found that OBP was higher during hypotension and lower during hypertension. A retrospective study by Wax and colleagues in anesthetized patients found that the BP values from OBP were higher than those recorded by IBP monitoring during periods of hypotension but lower during periods of hypertension [28]. In our study, although Figure 2 may suggest that the largest between-technique differences were outside normal BP values, there was no unequivocal correlation between these differences and BP values, but BP values detected either with auscultatory or oscillometric methods were often unpredictably very different from the real one. For this reason, we suggest that noninvasive techniques cannot be regarded as reliable alternatives to IBP.

The differences we reported may depend on the site where BP was measured. NIBP measurements were performed on the brachial artery, whereas IBP measurements were performed on the radial artery. It is known that IBP monitoring provides different BP values according to the site of detection and that brachial BP tends to be closer to central BP than radial BP [30–32]. However, several studies found that BP measurement in the brachial artery by both intra-arterial and auscultatory methods provided different results [27]. Moreover, comparisons of radial IBP with wrist or finger OBP did not produce uniform results, as expected [19, 22, 23, 33]. Unfortunately, in clinical practice decisions are often based on the BP values that are available, regardless of the method (NIBP or IBP) or the site (radial or brachial artery) where BP is detected. To overcome these limitations, a recent study from Wax and colleagues has shown that in a perioperative setting the concomitant use of OBP and IBP monitoring compared with IBP alone was associated with decreased use of transfusions, vasopressors, and antihypertensive drugs. The authors conclude that concomitant use of NIBP and IBP monitoring should be recommended to help interpret BP abnormalities and assist in clinical decision-making [28].

This study has some limitations. First of all, the patients included in this study differed in terms of their main diagnosis. However, data from previous studies have demonstrated that underlying diseases do not contribute to the differences between different methods [34]. Second, the formula used for the calculation of MAP with the auscultatory method may have been less accurate in either bradycardic or tachycardic patients, due to the length of the systole changing with the heart rate [35]. Unfortunately, there is no formula for MAP from the ABP method that adjusts for the heart rate. Third, we were able to include only a few patients with BP values outside the normal range; more research is needed in hypotensive and hypertensive patients, where the decision-making is particularly important but, at the same time, the vital information may be especially inaccurate.

## 5. Conclusions

ABP, OBP, and IBP are not based on the same physiological observation and measurement with different devices may not produce the same results. So it is very difficult to establish what is the “true” blood pressure, that is, the one on which to base clinical choices and derive data for calculating various scores. Even standardizing the technique and limiting interobserver variability, we found that both studied noninvasive methods (auscultatory and oscillometric) can be inaccurate among critically ill patients. Since in critically ill patients the importance of BP readings is often crucial, noninvasive techniques cannot be regarded as reliable alternatives to direct BP measurements. In settings where IBP monitoring is not possible and only noninvasive techniques are available, BP values detected by noninvasive methods may be randomly and unpredictably very different from the real one. In these cases, according to our findings, SAP values should be considered less reliable, while MAP appears to be the most reliable parameter, especially if assessed by the auscultatory method.

Since NIBP monitoring is normally available in every ICU, its use should be recommended in addition to IBP monitoring. If the data obtained by the two methods differ markedly and/or are not consistent with the patient's clinical condition, the operator should maintain a suspicious eye and check the reliability of the instruments, especially before undertaking any treatment. When IBP is not measured in the ICU for any reason, a comparison of ABP and OBP values is recommended.

## Figures and Tables

**Table 1:** Differences between patient systolic, diastolic, and mean arterial pressure taken with direct-invasive (IBP) and, respectively, with auscultatory-aneroid (ABP) and oscillometric automated (OBP) methods.

	Systolic	Diastolic	Mean
IBP			
Mean ± SD	134.4 ± 24.1	57.9 ± 12.3	81.5 ± 14.5
(median)	(134.5)	(57.0)	(79.5)
ABP			
Mean ± SD	124.7 ± 18.3	63.1 ± 12.9	83.7 ± 12.2
(median)	(125.0)	(62.0)	(82.0)
Difference (IBP − ABP)			
Mean ± SD	9.7 ± 13.8	−5.1 ± 7.1	−2.1 ± 7.1
(95% CI)	(7.0; 12.5)	(−6.5; −3.7)	(−3.6; −0.7)
*P* value	*P* < 0.0001	*P* < 0.0001	*P* = 0.0033

IBP			
Mean ± SD	134.2 ± 24.3	58.0 ± 12.7	81.6 ± 14.6
(median)	(134.0)	(57.0)	(80.0)
OBP			
Mean ± SD	123.4 ± 16.7	61.6 ± 11.9	76.2 ± 11.2
(median)	(125.0)	(60.5)	(75.5)
Difference (IBP − OBP)			
Mean ± SD	10.8 ± 14.9	−3.6 ± 6.0	5.4 ± 7.1
(95% CI)	(7.8; 13.8)	(−4.8; −2.4)	(4.0; 6.8)
*P* value	*P* < 0.0001	*P* < 0.0001	*P* < 0.0001

**Table 2:** BHS grade of agreement between noninvasive and direct-invasive methods: cumulative percentage of absolute difference (mmHg) between IBP and other studied methods (ABP and OBP). Grades are derived from percentages of readings within 5, 10, and 15 mmHg: to achieve a grade, all three percentages must be equal to or greater than the tabulated values. For example, to achieve the “A” grade, sixty percent of the measured BP values with IBP and ABP must be within 5 mmHg, 85% within 10 mmHg, and 95% within 15 mmHg. The limit of ≤20 mmHg does not belong to the BHS criteria and has been inserted to highlight in particular the poor agreement for SAP.

	≤5 mmHg	≤10 mmHg	≤15 mmHg	≤20 mmHg	BHS grade
IBP versus ABP					
Systolic	31%	53%	63%	77%	D (very poor)
Diastolic	51%	76%	94%	98%	B (good)
Mean	50%	85%	97%	99%	B (good)
IBP versus OBP					
Systolic	20%	40%	63%	74%	D (very poor)
Diastolic	62%	91%	96%	99%	A (very good)
Mean	40%	72%	96%	100%	C (poor)

IBP: invasive blood pressure; ABP: auscultatory-aneroid blood pressure; OBP: oscillometric automated blood pressure; BHS: British Hypertension Society.
